# Alcohol Craving in Heavy and Occasional Alcohol Drinkers After Cue Exposure in a Virtual Environment: The Role of the Sense of Presence

**DOI:** 10.3389/fnhum.2020.00124

**Published:** 2020-03-31

**Authors:** Jessica Simon, Anne-Marie Etienne, Stéphane Bouchard, Etienne Quertemont

**Affiliations:** ^1^Psychology and Neuroscience of Cognition—PsyNCogn, University of Liège, Liège, Belgium; ^2^Interfaculties Research Unit on Health and Society—URiSS, University of Liège, Liège, Belgium; ^3^Cyberpsychology Lab—Université du Québec en Outaouais, Gatineau, QC, Canada

**Keywords:** virtual reality, cue exposure, immersion, craving, sense of presence

## Abstract

The development of new technologies, and more specifically the opportunity to immerse participants in virtual controlled environments, provides a new ecological framework for researchers to study complex behaviors. This experiment aimed to compare post-immersion craving in occasional and heavy alcohol drinkers. Twenty-two occasional drinkers and eighteen heavy drinkers were recruited and immersed in a virtual bar, including alcoholic beverages. After the exposure, heavy drinkers reported a significantly higher craving than occasional drinkers. Post-immersion alcohol craving was significantly related to the levels of perceived ecological validity of the virtual environment. Finally, a moderation analysis suggested that the levels of craving more strongly increased with perceived ecological validity in heavy drinkers than in occasional drinkers. Therefore, the perceived ecological validity was an important experimental parameter to study craving in a virtual environment. These results further suggested that virtual reality might be a useful tool for both the scientific study of alcohol addiction and the treatment of alcohol dependence and relapse.

## Introduction

Alcohol craving is defined as the irrepressible desire to consume the substance and is considered as an important element in the development of alcoholism (Robinson and Berridge, 1993; Field and Cox, 2008) and alcohol relapse (Evren et al., 2010; Sinha et al., 2011). Many studies have investigated how various cues can increase alcohol craving and therefore to trigger alcohol relapse (Pericot-Valverde et al., 2015). However, most cue reactivity studies suffer from several limitations: the experimental paradigms in laboratory settings usually involve behaviors that are far from natural, often use simple stimuli (words or objects) on a computer screen, only allowing for a reduced complexity of behavioral interactions, therefore reducing the generalization of the results to real-life situations (Field and Cox, 2008; Field et al., 2016). The development of new technologies, and more specifically the ability to immerse participants in complex and multi-sensory virtual environments, brings a new ecological framework to study complex behavior while ensuring a high level of experimental control (Parsons, 2015). In recent years, some researchers have investigated the possibility of inducing alcohol craving by using virtual reality in a wide variety of samples. Particularly, previously published studies suggest that immersion in virtual alcohol-related environments enhances craving in participants with alcohol use disorders (Bordnick et al., 2008), and in abstinent alcohol-dependent patients (Lee et al., 2008). In non-clinical populations, Cho et al. (2008) also showed that a virtual alcohol-related environment can induce alcohol craving in non-dependent drinkers. To date, only one study has directly compared craving levels between two non-clinical samples of alcohol drinkers. In that study, binge drinkers reported higher levels of craving than non-binge drinkers after an immersion in virtual environments (kitchen and party) with alcohol-related cues (Ryan et al., 2010).

All these previous studies included either an assessment of subjective craving before immersion or repeated subjective craving measurements after each exposure to virtual environments. However, the self-reported assessment of craving requires participants to engage in introspection. As it is well established that self-monitoring alters the behavior being monitored (Perlmuter et al., 1983), such assessments before immersion in a virtual environment may create or modify the conscious experience of craving (Baker and Brandon, 1990) and therefore bias the results of the experiment. Furthermore, pre- and post-exposure measurements of craving expose to the risk of response-shift bias (Howard, 1980). Response-shift bias is a serious source of contamination of self-reported measures and occurs when participants recalibrate the meaning of their ratings after repeated requests of self-evaluation. Craving is a subjective affective state and the criterion used by participants to establish its assessment may change with time. Particularly, the pre-exposure craving measurement may change the assessment standard of the participant, i.e., his internal frame of reference, and result in inaccurate post-test ratings of craving (Rohs, 1999). Whenever such shifts occur, conventional self-reported pre- and post-exposure designs are unable to accurately assess the impact of craving. Additionally, pre-experimental assessment of craving can influence participants’ expectations about the objectives of the experiment and lead them to adjust their behavior to these expectations. To our knowledge, no study with exposure to virtual environments has yet attempted to minimize the impact of participants’ expectations on the assessment of craving.

The quality of immersion in virtual reality could affect self-reported craving. In particular, the reaction of people immersed in virtual reality involves the integration of multi-sensorial cues to activate emotions which are in turn related to the sense of presence (for a review of the issue see Diemer et al., 2015). The sense of presence describes the user’s feeling of really being *in* the virtual environment (Slater and Wilbur, 1997; Schubert et al., 2001). Particularly, the authors observed a positive correlation between the sense of presence and the emotional experience (Price and Anderson, 2007; Riva et al., 2007; Bouchard et al., 2008). Based on these observations, a higher sense of presence may be associated with higher levels of self-reported craving. Furthermore, the sense of presence might be either a mediator or a moderator of the effects of past alcohol experience on self-reported craving after an immersion in a virtual environment. The impact of the sense of presence as a mediator or as a moderator refers to two different explanations of how virtual reality can shape the behavior of participants. Following the “moderation” hypothesis, the sense of presence has a differential effect on self-reported craving according to the experience of alcohol consumption. In other words, a high sense of presence would significantly increase alcohol craving on average, but more strongly in heavy drinkers than in occasional drinkers. However, in a pure “moderation” hypothesis, heavy and occasional drinkers are not expected to differ in their sense of presence, even in an alcohol virtual environment (such as a bar). In statistical terms, the moderation translates into a significant alcohol group by presence interaction. According to the “mediation” hypothesis, the effects of alcohol history on self-reported craving would be mediated by the sense of presence. Previous alcohol experiences would significantly affect the sense of presence in an alcohol virtual environment, which in turn would affect alcohol craving. In such an explanation, heavy drinkers would have a higher sense of presence in a virtual environment related to alcohol because of their experience with alcohol and this would induce higher post-immersion craving than in occasional drinkers. In statistical terms, there would be a significant relationship between the levels of alcohol consumption in real life and the sense of presence in a virtual bar and when both variables are included in a multiple regression, the effects of alcohol consumption on post-immersion craving would become statistically non-significant. Finally, it is noteworthy that the two explanations are not necessarily mutually exclusive as the sense of presence might be both a partial mediator and moderator of the relationship between alcohol history and post-immersion craving.

In the present study, we immersed occasional and heavy alcohol drinkers in a virtual alcohol-related context without expectations about the real purpose of the experiment. To avoid experimental biases, alcohol subjective craving was not recorded before the immersion. We hypothesized that heavy drinkers would report higher levels of self-reported craving after the immersion than occasional drinkers. We then tested which dimensions of the sense of presence may explain the self-reported craving beyond alcohol consumption behaviors. Finally, we tested whether the sense of presence is a mediator or a moderator of the relationship between past alcohol experience (i.e., being heavy or occasional drinker) and post-immersion alcohol craving.

## Materials and Methods

### Participants

Eighty-four participants, between 18 and 35 years old, completed an online screening questionnaire including the Alcohol Use Disorders Identification Test [AUDIT (Saunders et al., 1993)]. Based on their AUDIT results, 40 young participants were recruited as occasional drinkers with a score of less than or equal to 7 (*n* = 21) or as heavy drinkers with a score greater than or equal to 11 (*n* = 18). Participants who never consumed alcohol were excluded from the study. These cut off scores for occasional and heavy drinkers were defined according to the recommendations of the literature (see below). One of the participants had to quit the experiment due to physical discomfort (cybersickness). The participants did not report having neurological or psychiatric problems and were free from drugs that could affect cognitive functioning, and reported being in good health.

### Questionnaires

Alcohol Use Disorders Identification Test [AUDIT (Saunders et al., 1993)]. The AUDIT questionnaire includes 10 multiple-choice items measuring alcohol consumption, alcohol dependence and alcohol-related problems. In the present experiment, a cut off score of 7 was used to characterize the occasional drinker group, also usually qualified as low-risk drinkers (Saunders et al., 1993; Babor et al., 2001). Conversely, a cut off score of 11 was set for the recruitment of participants in the heavy drinker group, also sometimes reported as problem drinkers (Fleming et al., 1991).

Craving. Craving was assessed after immersion in the virtual environment. Participants had to complete four 10 cm Visual Analogue Scales [VAS (Kreusch et al., 2017); adapted from the Alcohol Craving Questionnaire (Singleton et al., 1994)]. The VAS evaluated the expectancy for positive reinforcement (“Having a drink would make things just perfect”), the strength of craving (“How strong is your craving to drink alcohol”), the intent to consume an alcoholic beverage (“If I could drink alcohol now, I would drink it”), and the lack of control (“It would be hard to turn down a drink right now”). An overall craving score was calculated by adding the score of the four scales (maximum score = 40).

ITC-Sense of Presence Inventory [ITC-SOPI (Lessiter et al., 2001); French version translated by the Laboratoire de Cyberpsychologie de l’UQO]. The ITC-SOPI is a self-report questionnaire composed of 44 items, scored on a scale from 1 to 5 (1 = strongly disagree, 5 = strongly agree), focusing on the user’s experience of immersion in a virtual environment. Four dimensions are derived: the spatial presence (the sense of being physically within the displayed environment and the feeling of interacting and controlling objects), the engagement (the feeling of being psychologically involved), the ecological validity (the feeling of believability, realism and naturalness of the displayed environment), and the negative effects (the adverse physiological reactions).

The short version of the UPPS-P Impulsive Behavior Scale (Billieux et al., 2012). The UPPS is a short 20-item scale assessing impulsivity on five dimensions through a four-point Likert scale (1 = strongly disagree to 4 = strongly agree): positive urgency (to act rashly under conditions of positive affect), negative urgency (to act rashly under conditions of negative affect), lack of premeditation (the inability to remain focused on a task), and sensation seeking (the tendency and openness to enjoy exciting or dangerous activities).

The Trait scale of the State-Trait Anxiety Inventory [STAI (Spielberger, 1983)]. The trait subscale of the STAI assesses trait anxiety through 20 items. All items are rated on a 4-point scale (1 = “Almost Never” to 4 = “Almost Always”). Higher scores indicate greater anxiety. In this experiment, we used the trait anxiety, as many studies reported a relationship between anxiety and alcohol consumption (Gilpin et al., 2015).

### Virtual Environment and Material

**Figure 1:**
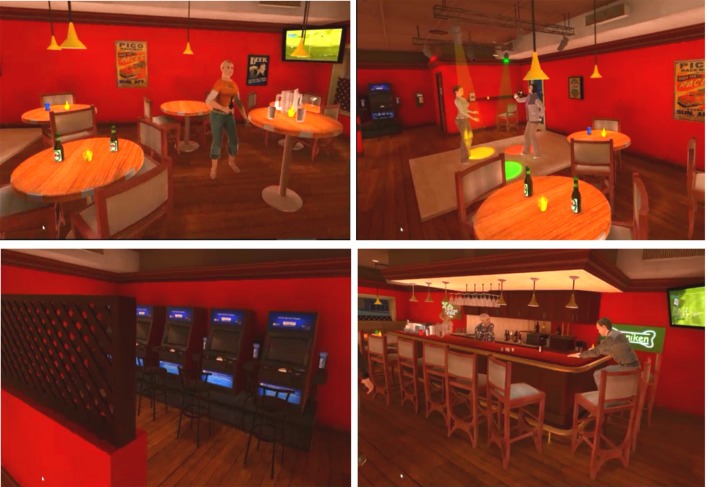
Screenshot of the virtual environment.

We used a virtual reality headset of the Oculus Rift brand. During the experiment, participants were immersed in a virtual bar where they heard background music, saw people dancing and/or drinking beer (Figure 1). This environment was originally developed by Professor Bouchard’s team (Bouchard and Robillard, 2015) and is the property of In Virtuo^1^. It includes different spaces: a counter, tables, a dance floor and one space dedicated to gambling. Bottles of alcohol (mainly beer) can be found in different places: several are placed on and behind the counter, two bottles of beer are arranged on a table and three characters hold a beer bottle in their hand.

### Design and Procedure

The study was approved by the Ethics Committee of the Faculty of Psychology of the University of Liège and was conducted following the ethical standards described in the Declaration of Helsinki (1964).

The participants of this study were recruited *via* word of mouth and *via* the website of the University of Liège. The announcement informed participants that this experiment was intended to assess the aesthetic qualities of a new virtual environment. Individuals interested in the study were invited to complete an online screening questionnaire including the AUDIT, the UPPS-P, and the STAI questionnaires. Participants meeting our inclusion criteria were invited to the laboratory for a session that lasted for about 1 h. They were tested one by one in a small quiet room. The experiment began with the signing of the consent form and with the collection of demographic information. They were informed that the immersion in virtual reality would take place in several parts, the final objective being to evaluate the quality of the environment that they would discover. After they had been explained the course of the experiment, the participants were invited to start the immersion. They had 1 min to become familiar with the technology and the use of the joystick to move around the environment. They were then invited to explore the four main parts of the virtual environment (the counter, the dance floor, the slot machines and the tables at the entrance) during a time-lapse from 30 s to 1 min each. The exploration order of these places was random across the participants. This procedure ensures that each participant fully explored the environment and received a minimum level of alcohol-cue exposure. In the third phase, the participants were instructed to freely explore the whole environment for a period of 2–3 min. When the 2 min were reached, they were asked whether they wanted to continue exploring the environment for an extra minute, or whether they wanted to start the fourth and final phase of the experience. Finally, the participants had the opportunity to re-explore a particular place of the environment for 2 min, although this was not included as a dependent variable in the experimental protocol and the time spent in the various parts of the virtual environment at this stage was not recorded. Once the time was up, participants removed the helmet and were invited to complete four scales evaluating craving and the ITC-SOPI questionnaire. Finally, the experimenter explained to the participants the real purpose of the study (i.e., debriefing) before asking them to sign a second informed consent.

### Statistical Analyses

Statistical analyses were performed using SAS software, Version 9.4 (SAS Institute Inc, 2013) and SPSS25.0 (SPSS Inc, 2017). All the analyses set the alpha level for statistical significance at 0.05.

In a first part of the analyses, differences between occasional and heavy drinkers were tested on the main demographic (gender, age), psychological/behavioral (STAI, UPPS dimensions, sense of presence) variables using Pearson’s chi-square test for independence or *t*-tests for independent groups (Table 1). Cohen’s *d* was used as a measure of effect size. Violation of the homogeneity of variance was checked using Fisher’s test. In case of severe violation, the Satterthwaite approximation was used instead of a *t*-test for independent groups.

**Table 1:** Demographic data, psychological characteristics and sense of presence in occasional and heavy drinker groups.

	Occasional drinkers	Heavy drinkers
Gender (F:M)	11:10	8:10
Age	24.62 (3.06)	23.72 (2.85)
AUDIT score***	3.81 (1.86)	15.22 (3.30)
STAI—Trait**	38.52 (7.17)	47.61 (10.86)
UPPS-P—Negative urgency*	7.52 (2.44)	9.72 (3.69)
UPPS-P—Positive urgency***	9.24 (2.07)	11.89 (2.32)
UPPS-P—Lack of premeditation*	6.90 (1.87)	8.39 (2.15)
UPPS-P—Lack of perseverance	7.05 (2.06)	7.78 (2.58)
UPPS-P—Sensation seeking	9.43 (2.34)	10.72 (2.47)
Presence—Spatial presence	2.78 (0.64)	3.10 (0.59)
Presence—Engagement	3.19 (0.43)	3.36 (0.74)
Presence—Ecological validity	2.89 (0.63)	3.26 (0.73)
Presence—Negative effects	2.93 (0.77)	3.26 (0.99)

Note. Standard deviations are in parentheses. *p < 0.05, **p < 0.01, ***p < 0.001.

To determine which of the four dimensions of the sense of presence is related to the score of craving, multiple regression analyses were computed with the craving score as the dependent variable and the four dimensions of presence derived from the ITC-SOPI as explanatory variables.

A Pearson correlation coefficient was calculated between the relevant dimensions of presence, as identified in the previous multiple regression analyses, and the craving scores separately for each group. The correlation coefficients were compared using the Fisher r –to- z transformation and discussed according to the effect size of the q-index derived according to Cohen’s classification (Cohen, 1988). A value less than 0.10 suggests no effect, a value between 0.10 and 0.30 represents a weak effect, a value between 0.30 and 0.50 evokes a medium effect and a value over 0.50, a large effect.

Finally, because the “ecological validity” dimension of the ITC-SOPI was the only statistically significant variable in the multiple regression analysis (Table 2), moderation and mediation analyses were performed using this subscale. In a moderation analysis, the effect of the moderator is statistically characterized as a significant interaction affecting the direction and/or the magnitude of the relationship between the dependent and independent variables. Therefore, we tested a linear hierarchic regression, including in the first block the alcohol drinker group and the ecological validity. In the second block, the interaction term between the group and the ecological validity was included. A moderating effect is defined as a significant improvement in the explanation with the inclusion of the interaction term. If the predictor and moderator become statistically non-significant with the introduction of the interaction term, the moderation is considered as complete. Similarly, a mediation model was performed based on Baron and Kenny’s recommendations (Baron and Kenny, 1986) to test whether the ecological validity mediates the relationship between alcohol consumption (drinker groups: occasional vs. heavy drinkers groups) and the self-reported craving. Evidence for a mediating effect of ecological validity requires fulfilling the following criteria: (1) the level of consumption (drinker groups) significantly explains the self-reported craving; (2) the level of consumption significantly explains the ecological validity. In other terms, the mean score of ecological validity significantly differs between groups; (3) ecological validity still significantly explains craving after controlling the model for the drinker group; and (4) when both the drinker group and the ecological validity are included together as predictors, the effect of the group is statistically reduced (partial mediation) or becomes statistically non-significant (full mediation). Finally, to ensure the significance of the mediation effect, we used the Sobel’s *test* (Sobel, 1982).

**Table 2:** Results of the multiple regression analysis with the craving score as the dependent variable and the four dimensions of the presence as explanatory variables.

Factor	Coefficient	SE	ß	*t*	*p*
Spatial presence	2.29	2.12	0.19	1.08	0.29
Engagement	1.51	2.09	0.12	0.72	0.47
Ecological validity	4.78	1.84	0.43	2.60	0.014
Negative effects	−0.25	1.19	−0.03	−0.21	0.84

## Results

**Figure 2:**
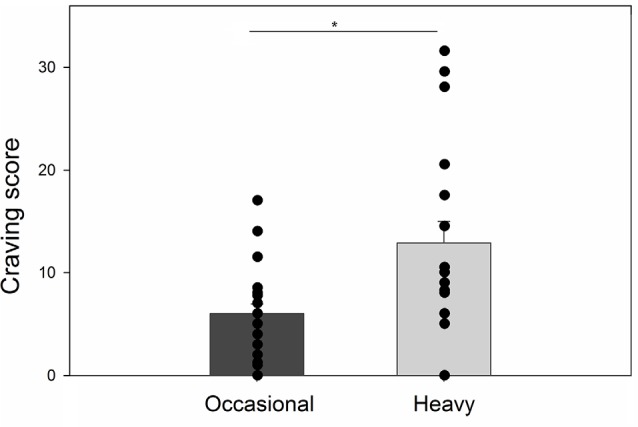
Average alcohol craving ratings for occasional and heavy drinkers and individual data points for each participant.

Groups did not differ in terms of gender ($\chi_{1}^{2}$ = 0.24, *p* = 0.75) and age (*t*_(37)_ = 0.94, *p* = 0.35). Heavy drinkers reported higher mean scores on the AUDIT questionnaire (*t*_(25.89)_ = 13.01, *p* < 0.001, *d* = 4.35) and, a higher level of trait anxiety (*t*_(37)_ = 3.12, *p* = 0.004, *d* = 1.00) than occasional drinkers. For the UPPS-P, the two groups significantly differed for the negative (*t*_(37)_ = 2.22, *p* = 0.03, *d* = 0.72) and the positive urgency (*t*_(37)_ = 3.77, *p* < 0.001, *d* = 1.21) and for the lack of premeditation (*t*_(37)_ = 2.31, *p* = 0.03, *d* = 0.74). In contrast, the mean difference between groups was not statistically significant for the lack of perseverance (*t*_(37)_ = 0.98, *p* = 0.33, *d* = 0.32) and for sensation seeking (*t*_(37)_ = 1.68, *p* = 0.10, *d* = 0.54; Table 1). Groups did not differ in terms of spatial presence (*t*_(37)_ = 1.61, *p* = 0.12, *d* = 0.52), engagement (*t*_(26.50)_ = 0.88, *p* = 0.39, *d* = 0.29), ecological validity (*t*_(37)_ = 1.70, *p* = 0.10, *d* = 0.55), and negative effects (*t*_(37)_ = 1.16, *p* = 0.25, *d* = 0.37). Finally, heavy drinkers showed higher levels of craving than occasional drinkers (*t*_(23.96)_ = 2.96, *p* = 0.007, *d* = 1.00) after the immersion (Figure 2).

Table 2 reports the results of the multiple regression analysis that was computed with the craving score as the dependent variable and the four dimensions of the presence as explanatory variables. The regression model accounted for 41% of the total variance in self-reported craving (*F*_(4,34)_ = 5.82; *p* = 0.001, R^2^ = 0.41; adjusted R^2^ = 0.34). Among the four dimensions of presence, only the perceived ecological validity was a significant predictor of self-reported craving (*p* = 0.014).

The correlation between the perceived ecological validity and the craving is only statistically significant for the heavy drinker group (*r* = 0.59, *p* = 0.01) but not for the occasional drinker group (*r* = 0.11, *p* = 0.65). The q index suggests that the difference between the correlations is of medium size (*q* = 0.43).

**Figure 3:**
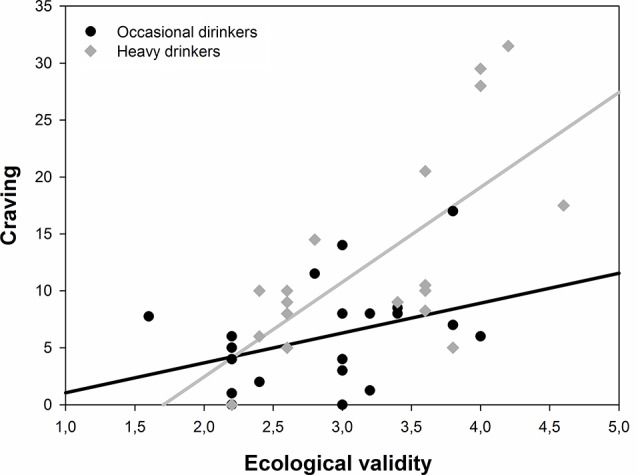
Prediction of the craving score in both drinker groups according to the levels of perceived ecological validity.

When testing the moderator effect of the perceived ecological validity of the environment on the relationship between alcohol drinker groups and self-reported craving, a complete moderation was obtained. The initial model (block 1) including the alcohol drinker group and the perceived ecological validity score was significant (*F*_(2,36)_ = 14.84, *p* < 0.001, R^2^ = 0.45, R^2^_adjusted_ = 0.42; drinker group: *β* = 0.37, *t* = 3.04, *p* = 0.004; perceived ecological validity: *β* = 0.52, *t* = 4.34, *p* < 0.001). The addition of the interaction term between the group and the ecological validity (block 2) significantly improved the model (*F*_(1,35)_ = 4.45, *p* = 0.042, ΔR^2^ = 0.06, ΔR^2^_adjusted_ = 0.05) and is associated with a loss of statistical significance of the two main effects [group (*t*_(35)_ = 1.49, *p* = 0.15), perceived ecological validity (*t*_(35)_ = −0.71, *p* = 0.49)]. As shown in Figure 3, the perceived ecological validity had a differential effect on self-reported craving according to the drinker group. Self-reported craving increased at a higher pace with perceived ecological validity in heavy drinkers than in occasional drinkers.

Finally, the mediation model was also tested to determine whether perceived ecological validity mediates the relationship between alcohol consumption (drinker groups) and self-reported craving. However, several of the basic assumptions of the mediation model were not met. Although occasional and heavy drinkers showed significant differences in their levels of self-reported craving, they did not significantly differ in the mean level of ecological validity (Table 1). Additionally, when both alcohol drinker groups and ecological validity were included as predictors in a multiple regression analysis with self-reported craving as the dependent variable, they both remained statistically significant [group (*t*_(35)_ = 2.47, *p* = 0.018), perceived ecological validity (*t*_(35)_ = 4.01, *p* < 0.001)]. Therefore, the mediation hypothesis is not supported by the present results.

## Discussion

The present study shows that heavy alcohol drinkers have higher craving scores than occasional drinkers after immersion in a virtual environment with alcohol-related cues, using an experimental design that reduces potential response and experimental biases associated with assessing craving before the immersion in virtual reality. Informing participants about the objectives of the study, or measuring craving before the immersion might induce different levels of craving based on alcohol consumption patterns, as previously suggested by Ryan et al. (2010). These authors observed different levels of craving between binge drinkers and non-binge drinkers after immersion in some specific virtual rooms (in the kitchen and the party room, but not in the bar or the argument room). The lack of statistical differences between the groups in the bar might be attributed to a lack of statistical power given their small sample size (15 binge drinkers and 8 non-binge drinkers).

Several studies suggested that the sense of presence affects the user experience in a virtual environment (Ferrer-García et al., 2010; Sylaiou et al., 2010; Ling et al., 2014; Brade et al., 2017). In previous studies, the sense of presence is related to the induction of fear in phobic participants. For example, Price et al. (2011) reported that the realness dimension of the IPQ (a 14-item self-report questionnaire assessing the sense of presence (Schubert et al., 2001) was related to the fear score in a social phobia sample. The present study suggests that alcohol craving is also sensitive to the realism of the virtual environment in interaction with the past alcohol consumption experience of participants. In the present study, we have tested two possible explanations of how the sense of presence might differentially affect alcohol craving in occasional and heavy drinkers. In the “mediation explanation,” the sense of presence mediates the effects of real-life alcohol consumption on post-immersion alcohol craving. In other words, with this explanation, heavy alcohol drinkers are expected to show a higher sense of presence in an alcohol-related virtual environment, which in turn would lead to higher levels of alcohol craving. This mediation explanation is not supported by the results of the present study. In particular, occasional and heavy drinkers did not significantly differ on the ecological validity dimension of the sense of presence. In the “moderation explanation,” the sense of presence modulates the effects of real-life alcohol consumption on post-immersion alcohol craving. In this second explanation, the sense of presence enhances post-immersion alcohol craving but more strongly (or only) in heavy alcohol drinkers. The results of the present study support the second explanation and agree with the complete moderation model. In heavy alcohol drinkers, alcohol craving is strongly related to the perceived ecological validity of the environment. In contrast, low alcohol craving is reported by occasional drinkers, whatever the levels of perceived ecological validity. In the present study, occasional and heavy drinkers also significantly differed in their levels of anxiety (Townshend and Duka, 2007) and impulsivity (de Wit, 2009) in agreement with previous results. Anxiety and impulsivity were not included as covariates in the regression models as they are distinctive characteristics of occasional and heavy drinkers and are related to their levels of craving. Controlling for these variables statistically removes a large part of the differences between occasional and heavy drinkers in their levels of reported craving.

Since ecological validity pertains to the level of perceived realism of the virtual environment, it can induce expectations regarding substance availability in the environment which in turn can generate craving (Field and Cox, 2008). The present results do not provide evidence that the other dimensions of the ITC-SOPI moderate the relationship between alcohol group and post-assessment craving. The spatial presence dimension assesses the feeling of being physically within the displayed environment and concerns technical aspects of the environment such as the feeling of interacting with objects. Understandably, this dimension is less related to alcohol craving than ecological validity. The third dimension, engagement, assesses the feeling of being psychologically involved and is a measure of a user’s interest in the content of the displayed environment. It is more surprising that this dimension is not related to the levels of post-immersion craving and this requires further studies. Finally, it was unlikely that the third dimension, negative effects or cybersickness, in a well-constructed virtual environment, would interact with the levels of post-immersion craving. Speculatively, it might have been expected that cybersickness would be negatively related to alcohol craving, but the present results do not provide evidence for such an effect. In conclusion, ecological validity, the feeling of believability, realism, and the naturalness of the displayed environment seems to be crucial for generating alcohol craving in heavy alcohol drinkers. While realism seems to be an important variable to take into account in drug addiction, the majority of similar previous studies used computer-generated virtual graphics with various levels of quality (Bordnick et al., 2008; Cho et al., 2008; Lee et al., 2008; Ryan et al., 2010) and often with a lack of graphical fidelity (Durl et al., 2018). In the field of drug addiction, future studies should, therefore, define the minimum threshold of realism and the best scenarios to maximize the induction of craving.

Virtual reality is a promising tool to improve our understanding of the mechanisms involved in drug and alcohol addiction. Field and Cox (2008) postulated that drug-related cues elicit an expectation of drug availability in the environment. In turn, availability expectations would increase attentional biases, such that the substance becomes the focus of attention, and would enhance the levels of craving. Furthermore, attentional biases and craving would keep mutually exciting relationships so that high craving would lead to focus attention on drug-related cues, which in turn, would further enhance craving. Such a “snowball effect” would be fundamental to the perpetuation of addiction and in the process of relapse (Cox et al., 2006). Virtual reality is an ideal technique to study the relationships between attentional biases and craving. As suggested by a previously published study (Ryan et al., 2010), virtual reality can induce craving in heavy drinkers. Furthermore, attentional biases might be measured in a virtual environment. The current experimental paradigms used to study attentional biases in addiction [the Addiction Stroop Task (Cox et al., 2006), the Visual Probe Task (Ehrman et al., 2002), the modified version for addiction of the Go/No-Go task (Kreusch et al., 2013) and the Stop-Signal (Kreusch et al., 2017)] suffer from serious limitations (Field and Cox, 2008; Field et al., 2016). In particular, these experimental tasks are generally far from natural behaviors, which could reduce the generalizability of the results to real-life situations. Such criticisms might be addressed by the implementation of eye-tracking technics to estimate attentional biases (Field and Christiansen, 2012; Christiansen et al., 2015) in a virtual environment.

Our results also suggest that virtual reality might be useful for the treatment of drug and alcohol addiction. As differential levels of craving between occasional and heavy drinkers are observable after immersion in an alcohol virtual environment, an obvious use of virtual reality is through desensitization treatments through exposure to alcohol-related cues. More broadly, virtual reality seems to be a promising tool in the development of new revalidation and prevention programs to generate lasting behavioral changes (Durl et al., 2018). Through the observation of patients in risky situations (in a restaurant, having a drink on a terrace, or spending an evening with friends), the therapist can better understand his behavior, help him to manage craving, improve his coping skills and teach new strategies to prevent relapse (Bordnick and Washburn, 2019). Virtual reality could also be used in the re-training of attentional biases in quasi-real situations, so that patients consistently learn to disengage their attention from the addiction-related cues. The present results further suggest that the realism of the virtual environment is an important factor to observe differential levels of craving between occasional and heavy drinkers after virtual immersion. As indicated by the moderation hypothesis, alcohol craving is significantly related to the perceived realism in heavy drinkers, whereas there is no such relationship in occasional drinkers. That no evidence for the mediation hypothesis was found also suggests that working with heavy drinkers does not ensure that a virtual alcohol environment will be perceived as a realist. Therefore the ecological validity of the virtual environment must be thoroughly assessed before any use in an experimental or clinical setting.

In the present experiment, we decided not to measure alcohol craving immediately before the immersion to avoid experimental biases (response bias and response-shift bias). Measuring alcohol craving immediately before immersion in the virtual environment would have informed participants that the study focused on the motivation to drink alcohol. There was a high risk of altering their behavior, for example drawing their attention towards alcohol-related cues. To conceal the real purpose of the study, the participants were told that they were taking part in a study assessing the aesthetic qualities of a virtual environment. However, there was a trade-off for avoiding these experimental biases: the lack of information regarding the levels of craving of participants before immersion. Although we cannot ensure that occasional and heavy drinkers had a similar initial level of craving before immersion, the differential correlations between the sense of presence and the craving in these two groups strongly suggest that craving in heavy drinkers is increased by the immersion in the virtual environment. Only in the heavy drinker group was the correlation between the sense of presence and the craving statistically significant. However, to definitively conclude that the increase in craving is due to the immersion in the alcohol-related virtual environment, it would be necessary to include a pre-immersion assessment of craving. So, future studies should directly test whether the assessment of craving before immersion in virtual reality alters the behavior of participants in the virtual environment and the levels of post-immersion craving. It would be also interesting to test whether the levels of pre-immersion craving are correlated with the sense of presence in the virtual environment.

In summary, the current study shows that heavy drinkers have higher levels of alcohol craving than occasional drinkers after immersion in a virtual environment with alcohol cues. Furthermore, the perceived realism of the virtual environment was shown to moderate the relationship between the levels of alcohol consumption in real life and post-immersion craving. Further studies are needed to investigate the relationship between craving and attentional biases measured in a virtual reality environment and to test the long-term efficacy of virtual reality as a treatment tool (Ghiţă and Gutiérrez-Maldonado, 2018; Segawa et al., 2020).

## Data Availability Statement

The datasets generated for this study are available on request to the corresponding author.

## Ethics Statement

The studies involving human participants were reviewed and approved by Ethics Committee of the Faculty of Psychology, Speech Therapy, and Educational Science of the University of Liège. The patients/participants provided their written informed consent to participate in this study.

## Author Contributions

EQ, A-ME, and SB: idea and implementation of experimental design and manuscript reviewing. SB: provision of the virtual environment. A-ME: virtual reality material and data collection. JS and EQ: statistical analyses. JS: manuscript writing.

## Conflict of Interest

SB is the president of and own equity in Cliniques et Développement In Virtuo, a university spin-off that uses virtual reality as part of its clinical services and distributes virtual environments. The terms of this arrangement have been reviewed and approved by the Université du Québec en Outaouais in accordance with its conflict of interest policies. The remaining authors declare that the research was conducted in the absence of any commercial or financial relationships that could be construed as a potential conflict of interest.

## Footnotes

^1^http://www.invirtuo.com
